# Selectivity and Sparseness in Randomly Connected Balanced Networks

**DOI:** 10.1371/journal.pone.0089992

**Published:** 2014-02-24

**Authors:** Cengiz Pehlevan, Haim Sompolinsky

**Affiliations:** 1 Swartz Program in Theoretical Neuroscience, Center for Brain Science, Harvard University, Cambridge, Massachusetts, United States of America; 2 Edmond and Lily Safra Center for Brain Sciences, The Hebrew University, Jerusalem, Israel; Indiana University, United States of America

## Abstract

Neurons in sensory cortex show stimulus selectivity and sparse population response, even in cases where no strong functionally specific structure in connectivity can be detected. This raises the question whether selectivity and sparseness can be generated and maintained in randomly connected networks. We consider a recurrent network of excitatory and inhibitory spiking neurons with random connectivity, driven by random projections from an input layer of stimulus selective neurons. In this architecture, the stimulus-to-stimulus and neuron-to-neuron modulation of total synaptic input is weak compared to the mean input. Surprisingly, we show that in the balanced state the network can still support high stimulus selectivity and sparse population response. In the balanced state, strong synapses amplify the variation in synaptic input and recurrent inhibition cancels the mean. Functional specificity in connectivity emerges due to the inhomogeneity caused by the generative statistical rule used to build the network. We further elucidate the mechanism behind and evaluate the effects of model parameters on population sparseness and stimulus selectivity. Network response to mixtures of stimuli is investigated. It is shown that a balanced state with unselective inhibition can be achieved with densely connected input to inhibitory population. Balanced networks exhibit the “paradoxical” effect: an increase in excitatory drive to inhibition leads to decreased inhibitory population firing rate. We compare and contrast selectivity and sparseness generated by the balanced network to randomly connected unbalanced networks. Finally, we discuss our results in light of experiments.

## Introduction

Across different modalities, sensory cortical neurons share a common response pattern: they respond sparsely, only a few neurons are active at any time, and selectively, a neuron responds to only few stimuli [1]. This has been observed in visual cortex [2]–[5], in olfactory cortex [6]–[8], in auditory cortex [9], [10] and in somatosensory cortex [11], [12].

On the theory side, many network models were proposed to explain the mechanism for generation and maintenance of selectivity and sparseness, especially for the orientation selectivity observed in visual cortex. Motivated by the existence of orientation columns observed in many species, these models predict that neurons with similar orientation preference are connected to each other with higher probability or with higher connection strengths [13]–[20]. Similar structured, functionally specific connectivity schemes have also been proposed in auditory cortex [21] and somatosensory cortex [22].

On the other hand, there are cases where sparse and selective responses are observed, while no significant preferential connectivity between cells with similar stimulus tuning can be detected experimentally. This is true in olfactory cortex [23]–[29] or in visual cortices of mice at eye-opening [30]. These examples motivate an important theoretical question: Can selectivity and sparseness be generated and maintained in randomly connected networks? Is it necessary for connection probabilities or strengths to be specifically chosen as a function of stimulus tuning?

To address this question, we consider a generic, randomly and sparsely connected network of excitatory and inhibitory spiking neurons, driven by random projections from an input layer. We require both the probability of connections and the strength of synapses between neurons to be unstructured. We found that despite its random architecture the network can exhibit high stimulus selectivity and sparse population response, if the network is in the balanced state [31], [32]. Interestingly, functional specificity in connectivity emerges in the network, even if the network is randomly connected. We elucidate the mechanism behind generation of selectivity and sparseness, discuss their dependence on model parameters, show that it is possible to achieve a balanced state with unselective inhibition, investigate the network’s response to mixtures of stimuli, discuss the paradoxical behavior of network response to changes in external drive to inhibitory population, and finally compare the network behavior to random but unbalanced networks.

Some of this work was presented in abstract form [33].

## Methods

### Model Network

#### Network architecture

We consider a randomly connected recurrent network of 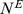 excitatory and 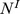 inhibitory leaky integrate-and-fire neurons driven by feedforward input from a population of 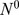 Poisson spiking neurons. Throughout the paper we denote population indices with capital letter superscripts, neuron indices with lower case subscripts and stimulus indices with Greek subscripts. For example, 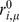 refers to the firing rate of the_
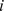
_^th^ input-layer neuron to 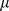 ^th^ stimulus.

In our network, a neuron of population 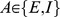 receives a synapse from a neuron of population 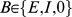 with probability_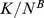,_ such that a postsynaptic neuron receives on average 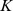 inputs from each presynaptic population. In the simulations presented, unless stated otherwise, 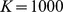, 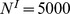, 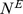 and 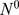 are 20000.

#### Activity of input neurons

Input neurons fire with Poisson statistics with a rate that depends on the stimulus. Each stimulus, indexed by subscript 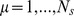, generates a random input firing rate pattern, 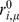. Each is drawn i.i.d., from a distribution density
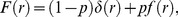
(1)where 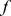 is an exponential distribution. For numerical stability we bound the exponential distribution to a maximum value 150 Hz. In the simulations, unless stated otherwise, we used 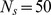 stimuli, 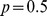 and a mean input population firing rate of 10.16 Hz.

#### Single-neuron dynamics

The subthreshold membrane potential dynamics of a neuron 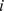 of population 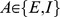, 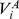, satisfies
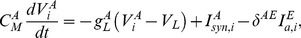
(2)where 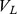 is the leak potential, 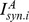 is the total synaptic current, and 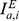 is an adaptation current which we only include for excitatory neurons. The dynamics of the adaptation current between spikes is given by



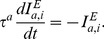
(3)When the voltage reaches 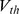 a spike is produced, voltage is reset to 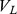, and the adaptation current is increased by 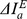.

Total synaptic input current is given by
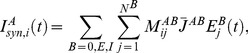
(4)where 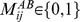 is the random connectivity matrix and 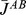 are the strengths of non-zero synapses. The postsynaptic currents 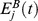 are linear sums of the contributions from individual presynaptic spikes given by a difference of exponentials,



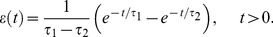
(5)Synaptic weights 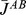 depend on 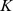, and are parameterized in two different ways:

*Weak synapses scenario:* 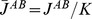, where 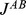 are independent of 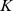
_,_ and *strong synapses or balanced network scenario:* 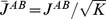.

#### Single neuron parameters

We used 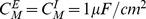, 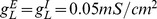 which led to 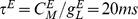, 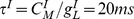. Other time constants were 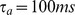, 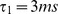, 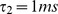. Leak potential was chosen to be 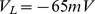, spiking threshold was 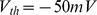, excitatory and inhibitory reversal potentials were 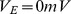 and 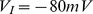 respectively. Synaptic coupling parameters for the strong synapses scenario were chosen to be 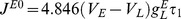, 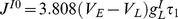, 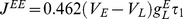,_
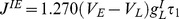
_, 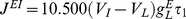, 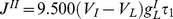. We note that 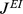 and 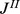 are negative. Adaptation current was increased at each spike by 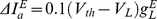.

#### Simulations

Model equations were integrated using a first order Euler method with 0.05 ms time steps. We verified the accuracy of results by repeating some of the simulations with a smaller time step of 0.025 ms. Simulations were generally run for 10 seconds, however depending on network firing rate, some simulations were run up to 100 seconds to gather better statistics.

### Analysis

#### Quantification of selectivity and sparseness

Both sparseness and selectivity can be related to the shape of the probability distribution of firing rates. The rate distribution of a population in response to a certain stimulus (population rate distribution) determines that population’s sparseness. Population rate distributions at different stimuli need not be identical to each other. The rate distribution of a single neuron across the whole set of stimuli (lifetime rate distribution) determines that neuron’s selectivity. Lifetime rate distributions of a population are in general different from each other and from population rate distributions [5], [34].

To quantify the sparseness of a population’s response to a stimulus 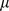, we define a sparseness index (SPI) [34]–[36]

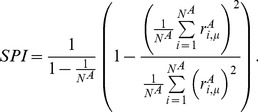
(6)


SPI varies between 0 (when all neurons respond identically) to 1 (when all but one neurons are silent). We defined a selectivity index (SLI) to quantify the selectivity of a single neuron as,
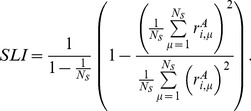
(7)


SLI ranges between 0 (when the neuron responds identically to all stimuli) to 1 (when the neuron responds only to a single stimulus).

In our model, in the input layer, population rate distributions at different stimuli (in the limit of infinite 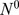) and neuron lifetime rate distributions (in the limit of infinite 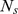) are all identical. SLI and SPI are both 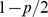, for finite 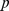 (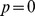 corresponds to all silent neurons). Importantly, for finite 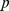, SLI and SPI do not depend on the parameter of the exponential distribution from which input population activity patterns are drawn from. Therefore, by adjusting this parameter one can alter mean input population firing rate without changing its selectivity or sparseness. Note that in this case the minimum selectivity that can be achieved in the input neurons is 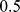, corresponding to the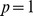 case.

#### Connection modulation index

Although connectivity is random, individual neurons do not respond equally to all stimuli. Hence, one can associate with each neuron a preferred stimulus, namely the stimulus that elicits maximum response from this neuron. To quantify the relation between stimulus selectivity and fluctuations in connections between neurons, we define the following Connection Modulation Index (CMI). Each synapse is classified into two groups: 1) synapses between neurons with similar preferred stimuli and 2) synapses between neurons with dissimilar preferred stimuli. A synapse falls into the first category if the preferred stimulus of the pre-synaptic neuron is one of the top 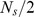 ranking stimuli of the post-synaptic neuron. Remaining synapses are classified into the second category. A probability is calculated for each category by dividing the number of synapses in that category by the total number of possible synapses in the network. Let 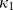 be the probability of a first category synapse and let 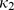 be the probability of a second category synapse. Then CMI is
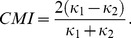
(8)


#### Selectivity of membrane potential

We defined a selectivity index for a neuron’s membrane potential, Voltage Modulation Index (VMI), as follows. Let 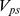 be the time-averaged membrane potential of a neuron at its preferred stimulus and let 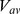 be the time-averaged membrane potential of a neuron averaged over all stimuli. Then,
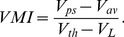
(9)


## Results

In this paper we focus on a generic network model with minimal assumptions about the nature of the underlying sensory computation. This is a randomly connected network of excitatory and inhibitory neurons, driven by random excitatory projections from an external excitatory population. Stimuli generate randomly drawn patterns of firing rates of the external population.

### Selectivity and Sparseness are Naturally Generated in the Balanced State

**Figure 1:**
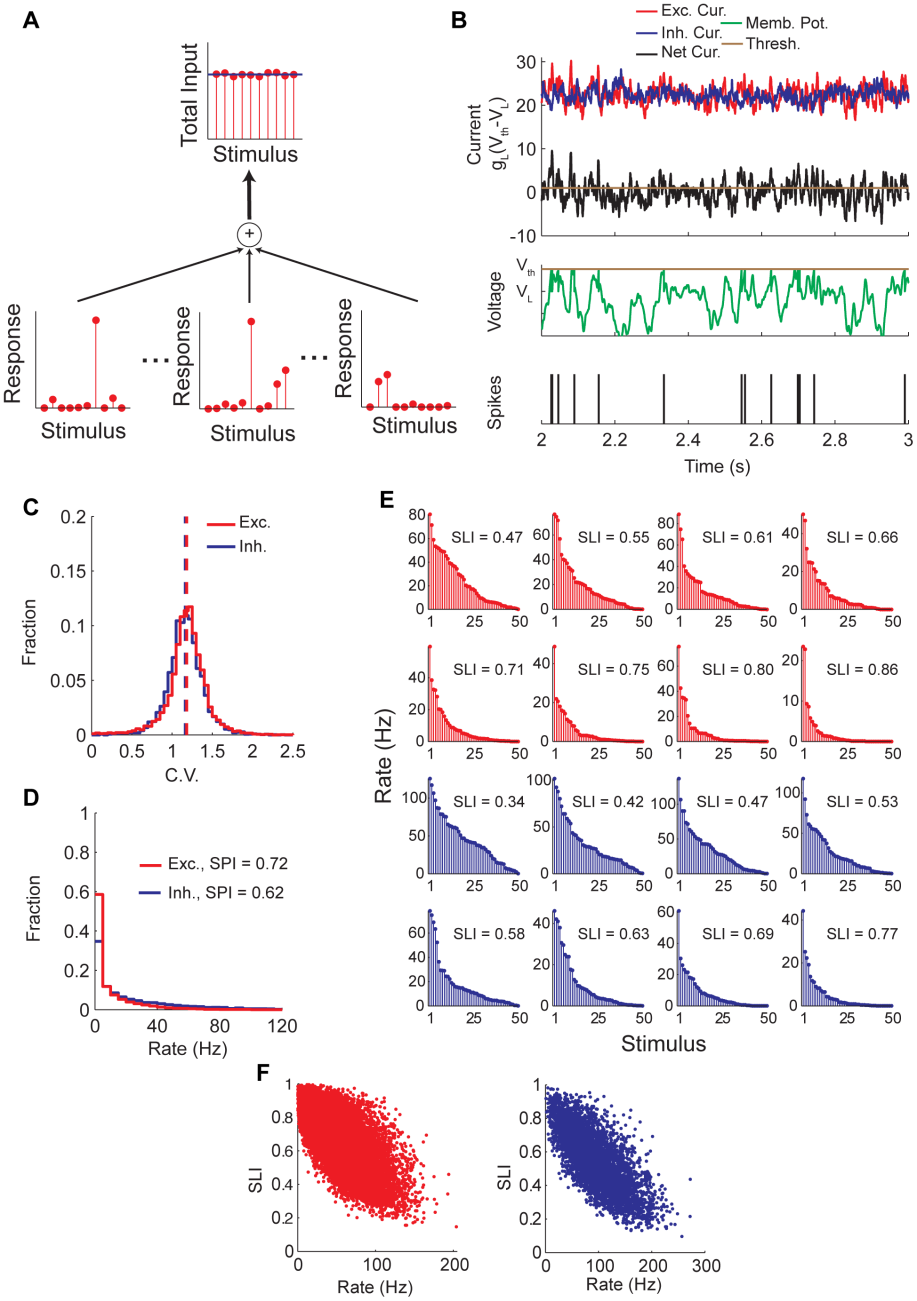
Randomly connected balanced network generates selective and sparse response. A) Selectivity is hard to sustain in a randomly connected network. Total input to a neuron has a small stimulus-to-stimulus variation compared to its mean, even if presynaptic neurons are highly selective. B) Example current and voltage traces. C) Distribution of ISI CVs for excitatory (red) and inhibitory (blue) populations. Vertical dashed lines show population means. D) Population rate distribution for excitatory (red) and inhibitory (blue) populations. Mean population firing rates are 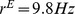, 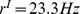. E) Magnitude ordered response profiles of example neurons. Red is for excitatory neurons, blue is for inhibitory. F) Scatter plot of neuron selectivity vs. response at preferred stimulus. Shown on left is the excitatory population and inhibitory population is on the right.

First we asked whether selectivity and sparseness could be generated in a network with random connectivity, where connection probability and the synaptic strength depend only on whether the presynaptic neuron is excitatory or inhibitory and not on any other feature. To see why this is challenging, consider neurons that are innervated by random projections from the same population of tuned neurons. When the expected number of projections, 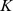, is large, the difference between the inputs to the neurons, or the difference between the inputs to each neuron induced by different stimuli, will be much smaller, by a factor of the order of 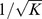, than the mean input (Fig. 1A). Thus, a special mechanism is needed to amplify this small variation to achieve heterogeneity and sparseness in firing rates.

We observed that this problem is solved naturally in a balanced network [31], [32]. A characteristic of balanced networks, and a key feature of our network, is the strong synapses: only 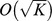 excitatory presynaptic neurons, out of on average 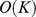, are needed to drive a neuron to firing. This is achieved by scaling synaptic weights by 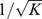
_,_ while the threshold is kept fixed (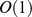). Sparse connectivity assures relatively small number of shared inputs between neurons and hence weakly correlated firing. Then, total synaptic current to a neuron generated by 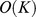 presynaptic neurons has an 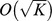 mean component and an 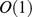 variable component. The mean would lead to large hyperpolarization or depolarization in the neuron, unless the excitatory input is nearly canceled by the inhibition. This cancelation does not require a fine-tuning of the network as one might expect. It was shown in [31], [32] that for a range of synaptic parameters the network settles into a balanced state, in which the mean excitation is almost precisely cancelled by the mean inhibition leaving an 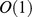 mean net input (Fig. 1B). Hence, the mean and the variable component of a neuron’s the total input current are of the same order of magnitude. Temporal fluctuations in the input current lead to strong temporal fluctuations in neuron spike times, with an interspike-interval (ISI) coefficient of variation (CV) around 1 (Fig. 1C). This mechanism was proposed to explain irregular spiking of cortical neurons [31], [32]. Here, we observe that the input current also has neuron-to-neuron and stimulus-to-stimulus variations, and as mentioned, these variations are comparable in magnitude to the mean. Hence, selectivity and sparseness can be maintained even when the mean number of connections per neuron is large (Fig. 1D–E). A similar observation was made in [37] for maintaining orientation selectivity in a model for a visual cortex without a functional map. We emphasize that the same mechanism also leads to sparse population response.

In Figure 1D, we show the histogram of single neuron’s firing rate responses to a single stimulus. The histograms are highly skewed. Quantifying sparseness by the Sparseness Index (SPI, see Methods), we found that inhibition has SPI = 0.62 and excitation has SPI = 0.72. Hence our random network is able to generate a significantly sparse response. Note that in our model, all stimuli are statistically identical; hence the sparseness is the same for all stimuli. On the other hand, the quenched heterogeneity in the connectivity across neurons implies that in the balanced state, the degree of stimulus selectivity varies between neurons. Fig. 1E shows representative tuning curves of single neuron firing rates. The distribution of Selectivity Index (SLI, see Methods) (also see below) is broad with mean 0.68 (st.d. 0.16) for excitatory and mean 0.56 (st.d. 0.17) for inhibitory populations.

### Weakly Responding Neurons are More Selective

As mentioned above, the stimulus selectivity varies considerably across the population. We asked whether this variation is related to the variation in the maximal firing rates. Fig. 1F shows a scatter plot of SLI vs. the firing rate of the neurons for their preferred stimuli, for both excitatory and inhibitory populations. Clearly there is a significant negative correlation (in this example, r = −0.69 for excitatory neurons and r = −0.73 for inhibitory neurons) between the SLI and maximal firing rates. The tendency of weakly responding neurons to have sharper selectivity can be explained by the spiking ‘iceberg’ effect, namely that for these neurons spike thresholds are higher than the mean membrane potential hence, they respond only to a narrow range of stimuli.

### Mechanism of Selectivity in the Balanced Network

**Figure 2:**
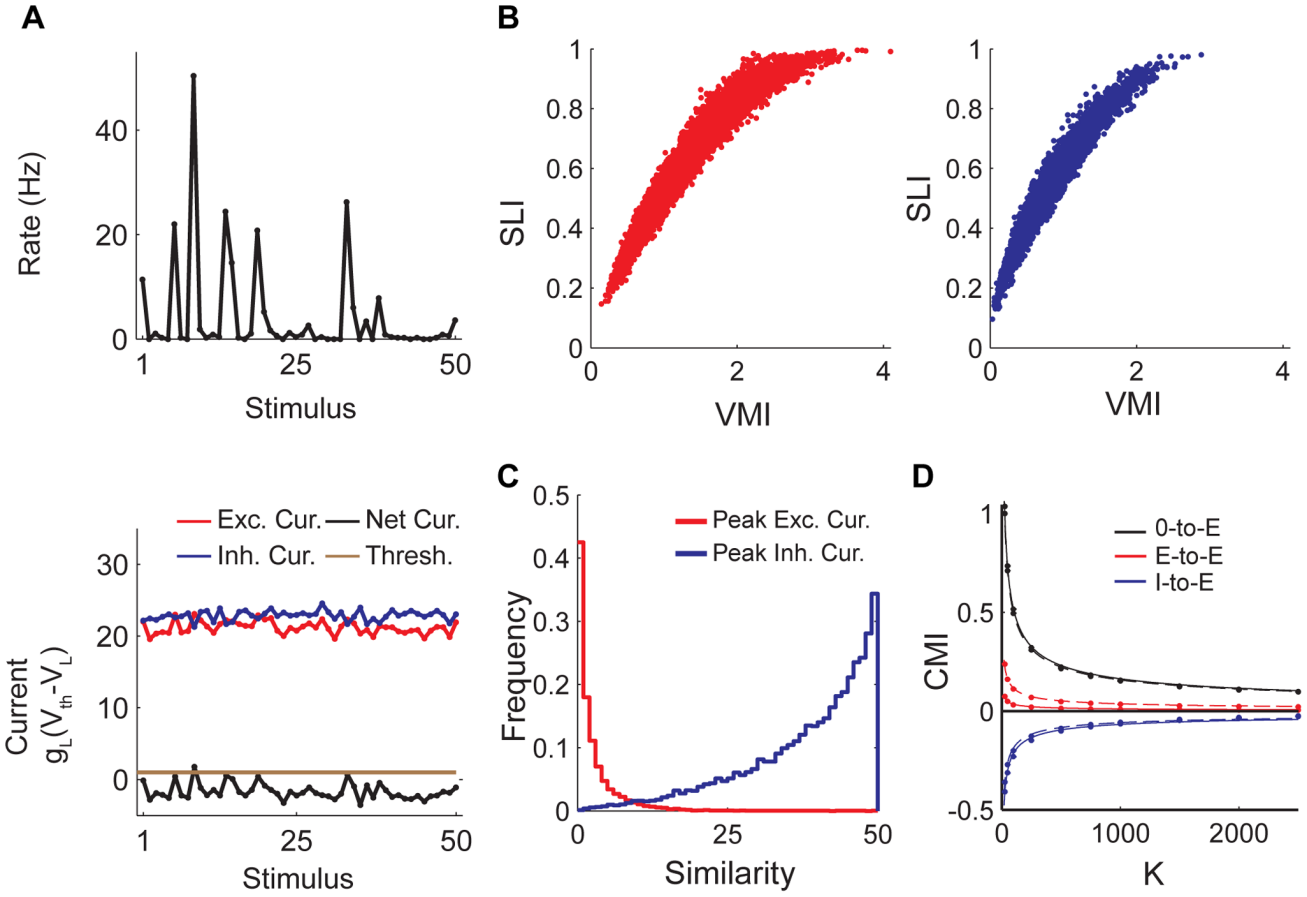
Selectivity and sparseness generation mechanism. A) On top is response to stimuli of an example neuron and on the bottom are time-averaged synaptic currents for corresponding stimuli. B) Scatter plot of neuron selectivity vs. voltage modulation index (VMI). Shown on left is the excitatory population and inhibitory population is on the right. C) Histogram of the similarity between peak current (excitatory current in red and inhibitory current in blue) and preferred stimulus for excitatory population. Similarity is defined as follows. For each neuron stimuli are ordered based on magnitude of response they elicit. Similarity is the difference in ranks of the stimulus that elicits peak excitatory or inhibitory current and the preferred stimulus, which is ranked 1. D) Dependence of connection modulation index (CMI, see Methods) on mean number of synapses a neuron receives from a particular population. Only E-to-E, I-to-E and 0-to-E type connections are shown. Lines are fits to the function 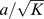 where 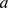 is the fitted parameter. Solid lines show CMI for our default parameters. Dotted lines correspond to a parameter set where 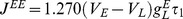. Excitatory network firing rate is kept at 10 Hz by adjusting the input mean firing rate at default input SPI = 0.75.

In the balanced network, even highly selective cells have broadly tuned inputs, as illustrated in the example of Fig. 2A, which is further sharpened by the spike threshold. There is a strong correlation between the selectivity of membrane potential and firing rate (r = 0.96 for excitatory neurons and r = 0.97 for inhibitory neurons) (Fig. 2B).

The selectivity in the net input is in general the combined effect of increased excitation in the preferred stimulus and decreased inhibition. Neurons tend to receive peak excitatory input at the preferred stimulus, while peak inhibitory input is more likely to be received at the least preferred stimulus (Fig. 2C).

### Emergence of Functional Specificity in Connectivity

To further elucidate the mechanism for tuning, we examined the correlation between the similarity in the preferred stimuli between pairs of neurons and their connection probability. We calculated the connection probability between excitatory neurons with similar preferred stimuli and compared it to the connection probability between excitatory neurons with dissimilar preferred stimuli, using the Connection Modulation Index (CMI) as defined in the Methods. CMI would be zero for no difference in connection probabilities, would be positive for bias towards neurons with similar preferred stimuli and negative otherwise. As depicted in Fig. 2D, the CMI is positive for the excitatory interactions (0->E and E->E) but is negative for the inhibitory interactions (I->E). Even if a random statistical rule was used to generate the network, functional specificity in connectivity emerged due to the inhomogeneity in this process. However, the magnitude of CMI falls with 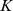
_,_ which denotes the mean number of synapses that a neuron receives from each population. In particular the CMI for the recurrent excitatory connections, E->E, is only a few percent for 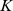 in the biologically relevant regime of few hundreds to thousands. These results indicate that the fluctuations in the feedforward connections provide the main source of selectivity in the output layer, while the recurrent excitatory and inhibitory connections mainly play the role of balancing the mean input.

### Selectivity and Sparseness Depend Only on the First Two Moments of the Input Population Activity

**Figure 3:**
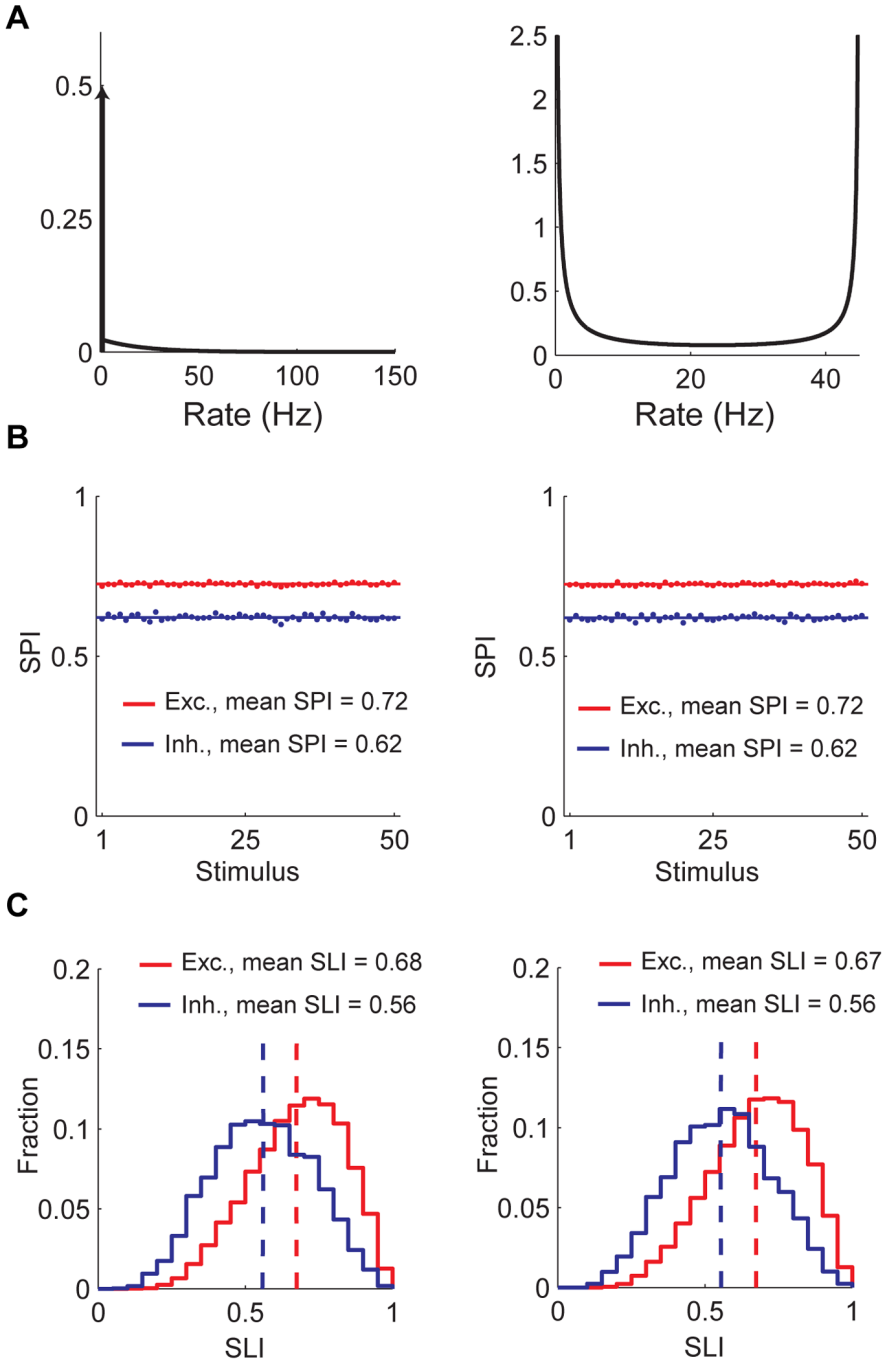
Only first two moments of the input activity determine network response. On the left is the balanced network with our default input population activity, consisting of a delta function at the origin with weight 0.5 and an exponential tail. On the right is the same network driven with another ensemble of stimuli, whose first two moments are matched to the default activity. Here, the response distribution is given by a beta distribution (with maximal firing rate 45 Hz), where its two parameters are chosen to match the mean and variance of the distribution in A. B) Population sparseness for different stimuli. C) SLI histograms.

We asked what features of the input rate distributions determine the network behavior. Due to the random connectivity and large number of synapses, both neuron-to-neuron and stimulus-to-stimulus variations of synaptic currents in the network are well approximated by a Gaussian distribution. This fact has been used in analytical treatments of balanced networks to come up with statistical descriptions of network activity [13], [14], [31], [37]. Therefore, only the first two moments of the input population rate distribution and lifetime rate distributions should affect the network’s operation. We tested this observation by using a different ensemble of activity patterns at the input layer than the default one while matching first two moments of the two ensembles (Fig. 3A). As expected, network SLI and SPI distributions for both stimulus ensembles are identical (Fig. 3B,C). Note also that in our model all stimuli are statistically identical, hence the rate distribution (and in particular the SPI) is the same for all stimuli (Fig. 3B).

### Selectivity and Sparseness Vary with Stimulus Intensity

**Figure 4:**
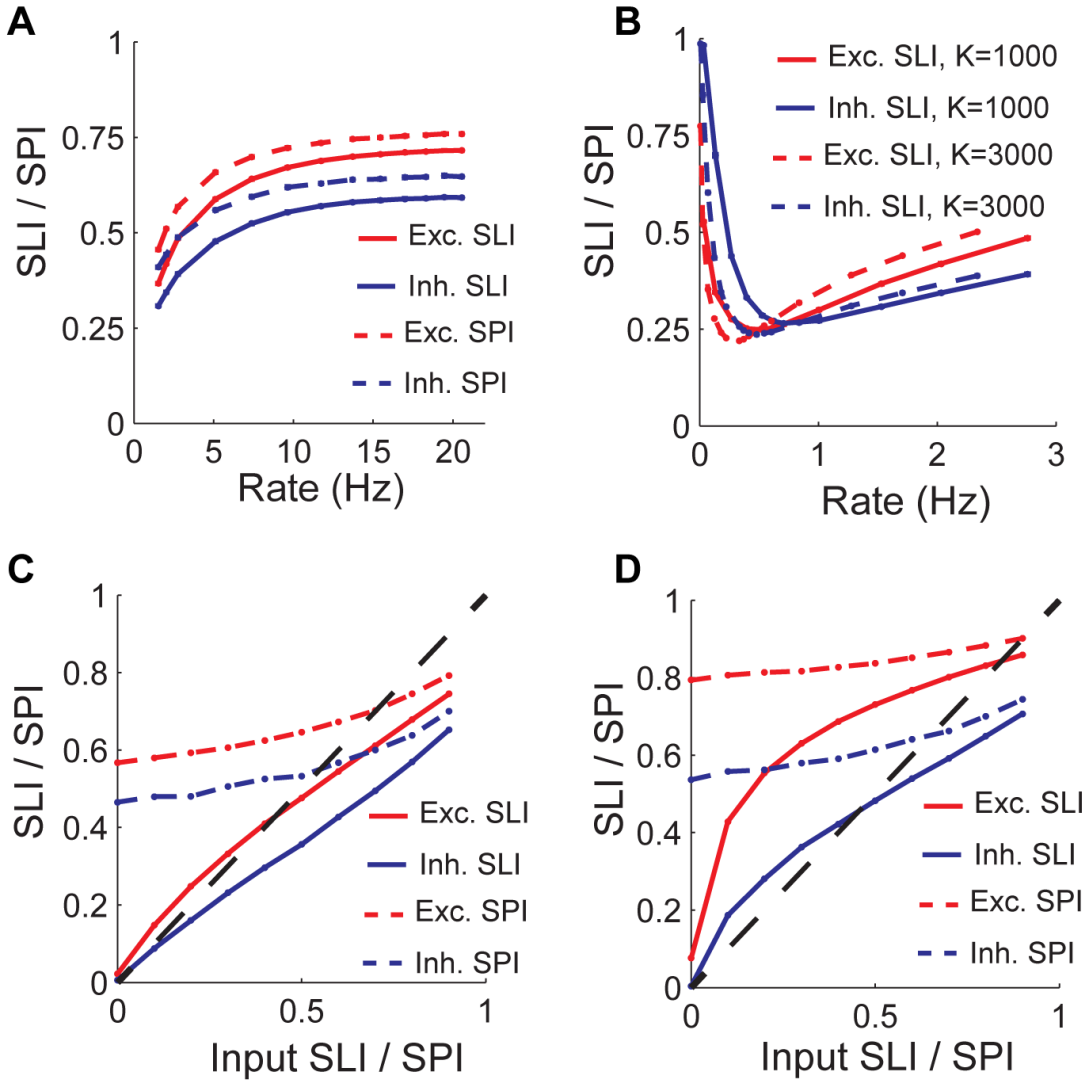
Dependence of selectivity and sparseness on input. A) and B) Selectivity and sparseness as a function of excitatory network firing rate. Mean input rate is modulated keeping its sparseness fixed. C) Selectivity and sparseness as a function of input sparseness. Input mean firing rate adjusted so that excitatory network fires at 10 Hz at default input SPI = 0.75. To achieve less sparse input population rate distributions than the minimum sparseness allowed by our default stimulus ensemble, we set 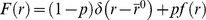, where 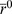 is the mean of 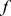, and therefore 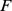. SLI and SPI are now given by 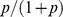. Note that numerically calculating 0 for SLI is impossible due to finite network simulation time. Trial-to-trial variability of Poisson-like firing neurons will lead to a residual selectivity even if the input is unselective, which will vanish only in the limit of infinite simulation time. In our simulations, we increased simulation time as needed to minimize this effect, especially for low input selectivity. D) Same as in C) but synaptic weight 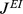 is changed from its default value to 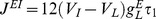.

Dependence of sensory representations to stimulus intensity has been a subject of many studies, e.g. [4], [38]–[42]. We modeled a change in stimulus intensity as a change in the mean firing rate of the input population (and the concomitant change in the rate of the output populations) without changing the shape of the rate distribution, i.e., with fixed input selectivity and sparseness (see Methods). We found that the selectivity and sparseness generated in the network decrease with decreasing the input rate, except for very low rates (Fig. 4A). Interestingly, this effect is opposite to what is expected from the above-mentioned ‘iceberg’ effect. The reason for this can be understood from a theoretical analysis of balanced networks in the low rate limit, given in [31]. It was shown that in the limit of infinite 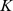, as the network rate decreases, the magnitude of the quenched neuron-to-neuron variation in the inputs decreases faster relative to the magnitude of stochastic fluctuations, which become the dominant drive to raise the voltage above spike threshold. Then, in this limit SPI should go to 0. A similar argument could be given for stimulus-to-stimulus variations and SLI. However for finite 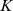 this is not the case. At a very low firing rate (of the order of 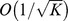) the mean number of input spikes to a neuron is so small that the system is no longer in the balanced state and the temporal fluctuations are small. In this regime the selectivity and sparseness increases to high values with decreasing mean rate (Fig. 4B), reflecting the fact that the spiking activity is dominated by the large static depolarization caused by a rare stimulus.

### Selectivity and Sparseness Increase Monotonically with Sparseness of Input

We examined the relation between the sparseness and selectivity of the input layer activity patterns and that of the output layers. For this purpose we have varied the ratio of the standard deviation of the rate distribution over its mean, resulting in changes in the SLI and SPI (recall that at the input layer SLI and SPI are identical) while the mean firing rate is held fixed. Note that this manipulation completes the study of the dependence of balanced network operation on input layer activity, since only first two moments of the input layer activation statistics is important for the balanced network’s operation and we already discussed the variation with respect to the mean with SLI and SPI kept fixed.

In Fig. 4C we plot network selectivity and sparseness as a function of input population selectivity. We observe that selectivity and sparseness in the network grow with increasing input selectivity. The results also demonstrate that SLI and SPI differ significantly when the input is nonselective (namely all stimuli activate uniformly all input neurons). In this limit all stimuli will elicit the same response for each output neuron, hence SLI = 0. On the other hand, the network can still generate significantly sparse response (i.e., SPI is high), because randomness in connection probability creates heterogeneity in the number of synapses a neuron receives, which in turn leads to sparseness even if all presynaptic input neurons fire at the same rate.

To emphasize this last point, we simulated another randomly connected network with nonselective input. This network had identical parameters, except that number of synapses a neuron receives from each population was constrained to be exactly 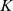. In this case both SLI and SPI were 0, confirming our point.

In Fig. 4C, which is based on our default parameter set, the selectivity of the output layer is almost always lower from the input one, implying lack of amplification of stimulus selectivity by the network feedforward and recurrent connections. This however depends on the precise values of these connections. For other parameter sets, such as shown in Fig. 4D, SLI of the excitatory population may be larger than the input SLI. In this example, inhibitory synapses to excitatory neurons are strengthened leading to increased inhibitory population rate, which is positively correlated with selectivity, as discussed in more detail below. Also, it is possible to find cases in which the inhibitory selectivity or sparseness is larger than the excitatory one, implying that in the balanced network the relative selectivities of the three populations vary with the specific values of the synaptic strength.

### Selectivity and Sparseness Generated in the Balanced State are Robust to Changes in Mean Number of Connections

**Figure 5:**
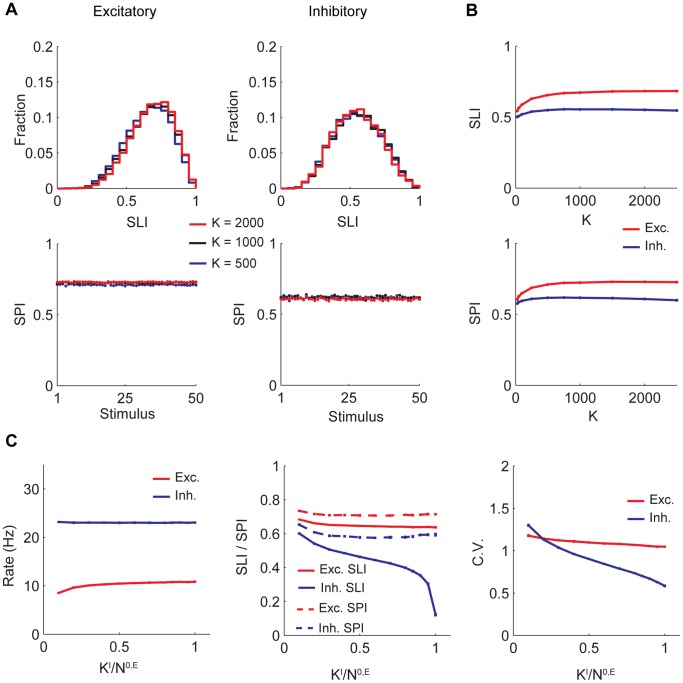
Selectivity and sparseness are robust to changes in mean number of connections. A) SPI for different stimuli and SLI histograms for excitatory and inhibitory populations at 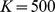, 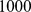 and 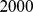. B) Top: Population averaged SLI as a function of 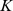. Bottom: Stimulus averaged SPI as a function of 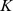. Excitatory population firing rate was kept at 10 Hz for A) and B). C) Left: Population firing rates vs. density of E->I and 0->I connectivity. Middle: Population averaged SLI and SPI vs. density of E->I and 0->I connectivity. Right: Population averaged ISI CV vs. density of E->I and 0->I connectivity. In these figures 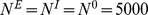 and 0->I and E->I synaptic strengths are scaled by 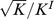 where 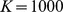. Mean input layer population firing rate was 10.18 Hz.

Next we asked whether the selectivity and sparseness generated in the network are robust to changes in_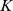._ In the balanced network, this dependence should be weak as long as 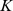
stays small compared to the network size, since the variation in input current relative to the mean is 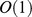 even for large 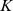 [31], [32], [37]. This expectation is borne out in the numerical results of Fig. 5A, which show that the SLI histograms and SPI curves for excitatory population at 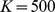, 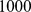 and 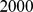, are very similar. Fig. 5B shows that both the mean selectivity and the sparseness are almost independent of 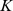 except for a slight decrease in their values (for the excitatory population) when 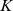 drops below 200.

### Densely Connected Input to Inhibitory Population Diminishes Inhibitory Selectivity

Inhibitory neurons in our model show high selectivity, however experiments report both selective and unselective interneurons in sensory cortex [7], [43]–[45]. Here we show that it is possible to achieve a balanced state with an unselective inhibitory population by increasing the density of connections to inhibitory neurons from the input (0->I) and the excitatory (E->I) populations, but keeping inhibitory to inhibitory (I->I) connectivity sparse. We denote the mean number of 0->I and E->I synapses per inhibitory neuron by 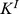. We scaled the strength of these connections so that the mean input to inhibitory neurons does not change. In Fig. 5C, we vary 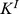 from 10% of the excitatory and input population network size to 100%. As 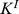 increases, a large decrease in inhibitory population selectivity is observed but inhibitory sparseness is mildly affected (Fig. 5C middle). The decreases in excitatory population selectivity and sparseness are also small. The limit of all-to-all 0->I and E->I connectivity provides an understanding of these effects. In this limit, 0->I and E->I inputs to each inhibitory neuron are proportional to corresponding presynaptic population’s average firing rate. There are no stimulus-to-stimulus or neuron-to-neuron variations in this input. Sparse connectivity within the inhibitory population causes neuron-to-neuron variations and temporal irregularity in the inhibitory responses. Therefore, inhibitory neurons show no selectivity but exhibit sparseness, which is reduced due to the disappearing variations in E->I and 0->I synaptic currents. The reductions in selectivity and sparseness of the inhibitory population lead to the (small) reductions in excitatory selectivity and sparseness.

We also observe a decrease in the CV of ISIs in the inhibitory population as 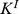 increases (Fig. 5C right). This can be explained by the reduction in temporal variability in the E->I and 0->I synaptic currents due to averaging from a larger pool of presynaptic neurons.

### Synaptic Strengths have a Modest Effect on Selectivity and Sparseness

**Figure 6:**
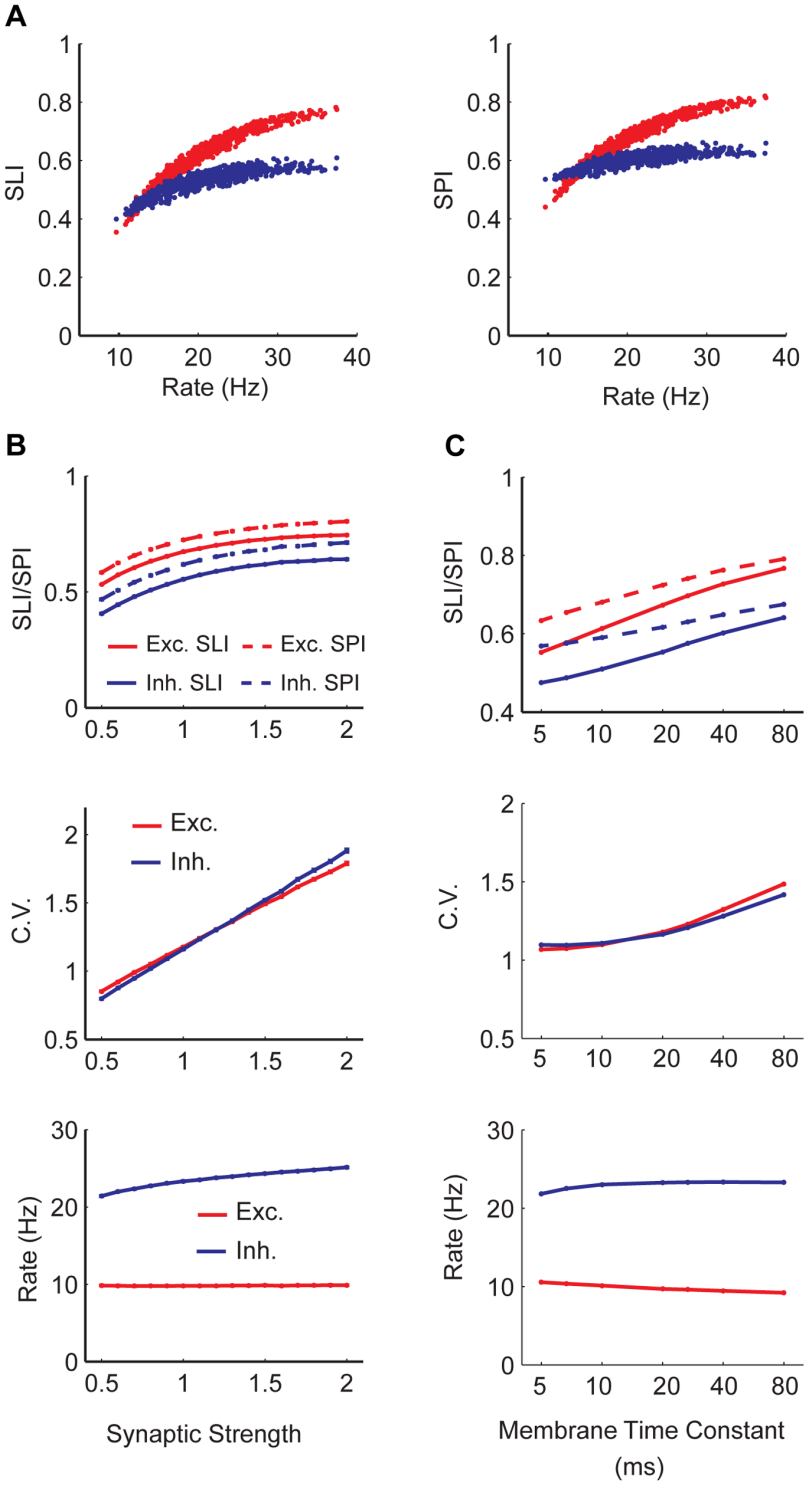
Dependence of selectivity and sparseness on synaptic weights and membrane time constants. A) Population averaged SLI and stimulus averaged SPI for excitatory (red) and inhibitory (blue) populations in 1000 networks with randomly generated synaptic weights within a 10% range of our default parameters. Mean input rate was adjusted to keep the mean rate of the excitatory neurons at 10 Hz and mean input SLI was kept fixed. B) All synaptic weights are simultaneously scaled with a multiplicative constant, which we call the synaptic strength. 1 corresponds to original parameters. Top: SLI and SPI as a function of synaptic strength. Middle: ISI CV averages for excitatory and inhibitory populations. Bottom. Population firing rate. C) Membrane time constants are varied from 5 ms to 80 ms. Same quantities are plotted as in B.

The parameters denoting synaptic strengths within and between populations form a high dimensional space, which is hard to explore exhaustively. To demonstrate the effect of changing synaptic parameters, we simulated 1000 networks where all synaptic weights were varied randomly within a 10% range of our default parameters. In all cases, the input SLI was kept fixed and the mean input rate was adjusted to keep the mean rate of the excitatory neurons at 10 Hz. Fig. 6A shows the scatter plot of the resultant values of SLI and SPI against the mean firing rate of the inhibitory population. Network selectivity and sparseness are modestly affected by changes in synaptic weights. As can be seen, the main factor that controls the variability in the selectivity and sparseness of the population is the increase in the inhibitory activity, which is positively correlated with increase in selectivity. For a fixed value of the inhibitory firing rate, the changes in the selectivity and sparseness are small.

We also scaled all synaptic couplings simultaneously keeping input rate and selectivity fixed. We found that increased overall synaptic strength leads to increased selectivity and sparseness (Fig. 6B). Neurons tended to fire with higher CV with increased overall synaptic strength. The changes in excitatory and inhibitory network firing rates were small.

### Selectivity and Sparseness Decrease with Shorter Membrane Time Constant

Next, we studied the effect of membrane time constant on network selectivity. Both excitatory and inhibitory neuron membrane time constants were varied simultaneously (Fig. 6C). We found that shorter membrane time constants lead to decreased selectivity and sparseness. Neurons fired with decreased CV. The changes in excitatory and inhibitory network firing rates were small.

### Network Response to Mixtures of Stimuli Shows Strong Suppression

**Figure 7:**
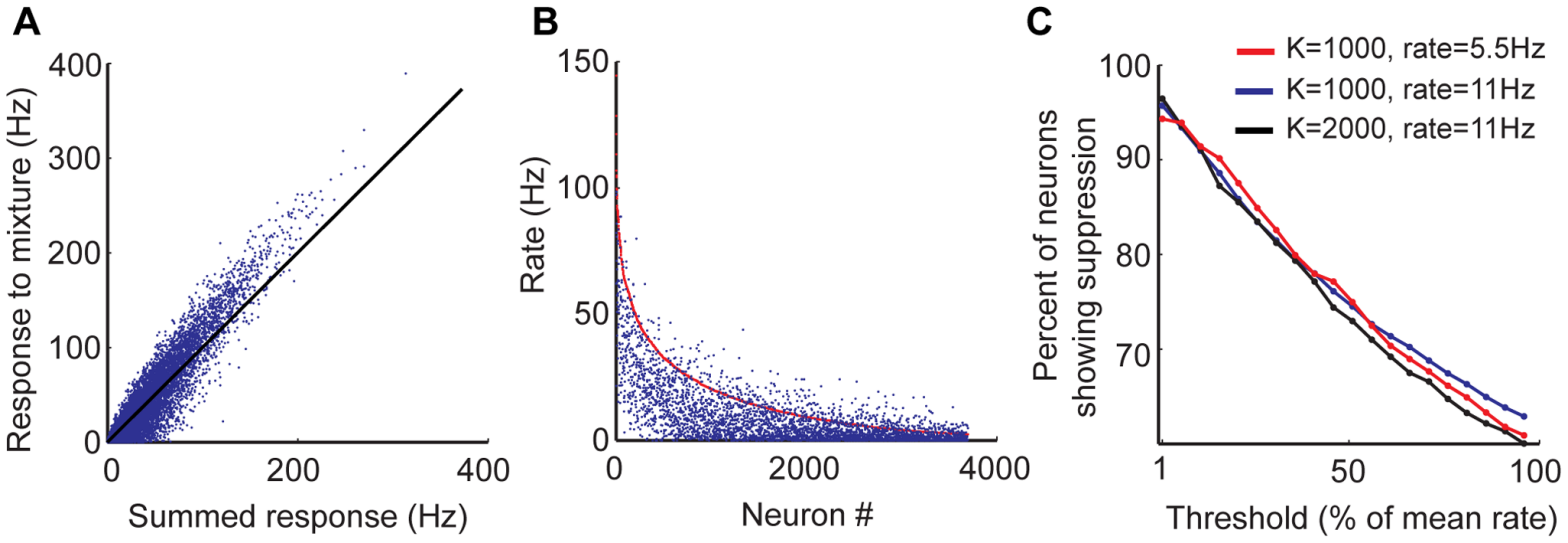
Network response to mixtures of stimuli. A) Scatter plot of excitatory responses to a mixture of two stimuli versus the sum of the responses to individual stimuli. Black line is the diagonal. Mean input layer population firing rate was 12.2 Hz for a single stimulus, leading to 11 Hz excitatory population firing rate. B) Mixture responses of neurons that do not respond to one of the component stimuli but respond to the second stimulus. Null response is defined as firing rate lower than 20% of the mean firing rate (in this case, the threshold is 2.2 Hz). Neurons are sorted by the magnitude of their response to the second stimulus. In red is the response to the second stimulus; in blue is the response to the mixture of two stimuli. A neuron is said to show suppression effect if the response to the mixture is less than response to the second stimulus. C) Ratio of the number of excitatory neurons showing suppression effect to the total number of excitatory neurons that respond only to one stimulus, as a function of the null-response threshold. The threshold is shown as a fraction of the mean excitatory firing rate. This curve is plotted for several connectivity numbers and mean rates.

How does the balanced network respond to mixtures of stimuli? Assuming that input layer responses are additive, we presented the network with a mixture of two stimuli and compared the resultant response to the response of the network to the individual stimuli. It is known that mean firing rate of a balanced network changes linearly with mean input firing rate [31], [32], leading to a linear population response to a mixture. However, when individual neurons are inspected, we observed that neurons with low firing rates show sublinear response but neurons with high firing rate show supralinear response (Fig. 7A). The observation of sublinear response for neurons with low firing rates led us to investigate if there is a suppressive effect in responses to mixtures. When we examined the pool of neurons that respond to only one of the stimuli (where null-response is defined as firing below 20% of the mean firing rate), we found that a large majority of these neurons showed significantly reduced firing rates when presented with the mixture (Fig. 7B). This effect was robust to network firing rate or density of connectivity. Although the quantitative effect depends on the threshold defining non-response, the suppression effect is significant for a wide range of the threshold (Fig. 7C).

### Paradoxical Behavior of Network Response to Changes in Inhibitory Activity

Networks stabilized by inhibition are known to exhibit a “paradoxical” effect when input to inhibitory cells is altered [46], [47]. Increasing the excitatory input to inhibitory cells causes a decrease in the firing rates of both excitatory and inhibitory populations, and conversely decreasing excitatory input causes an increase in inhibitory and excitatory firing rates. Balanced networks, being inhibition stabilized, also show this behavior. To see this, suppose external excitatory input to inhibitory neurons is increased. The change in the activity of excitatory and inhibitory networks should be in the same direction (both increase or both decrease) for balancing to be sustained in the input to excitatory population. Detailed analytical study of balanced networks [13], [31] shows that elimination of unbalanced states requires the network to be in a parameter regime (which is the case considered here) in which activity of both populations would decrease. The opposite is also true for decreasing external excitatory input to inhibitory neurons. For example, for a balanced network firing at about 5 Hz mean excitatory rate and 10 Hz mean inhibitory rate, elimination of all external input to the inhibitory neurons caused a drastic increase in the network response to rates above 50 Hz for both populations.

### Random Networks with Weak Synapses

**Figure 8:**
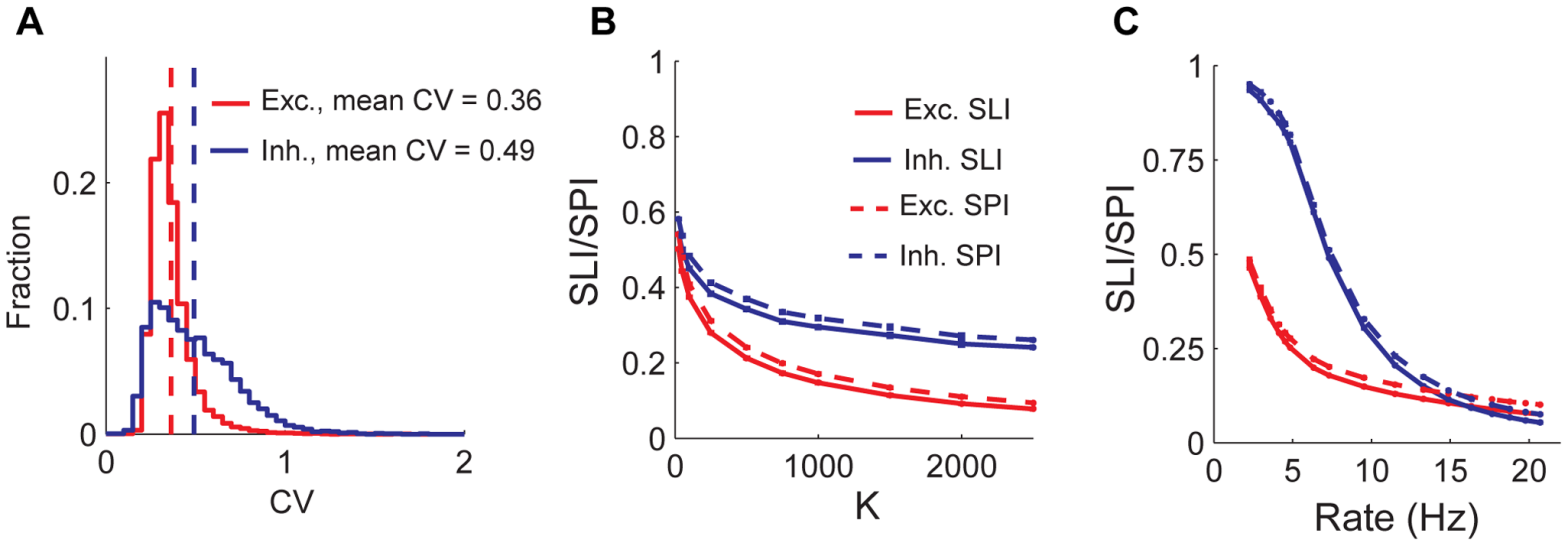
Selectivity and sparseness in a random network with weak synapses. A) Distribution of ISI CVs for excitatory (red) and inhibitory (blue) populations. Vertical dashed lines show population means. Excitatory population fires 9.6 Hz while the inhibitory population fires at 9.1 Hz. B) Population averaged SLI (solid lines) and stimulus averaged SPI (dashed lines) as a function of 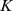. Excitatory population firing rate is kept fixed at 10 Hz by adjusting the mean input firing rate. C) Population averaged SLI (solid lines) and stimulus averaged SPI (dashed lines) as a function of excitatory population rate. For our simulations with weak synapses, synaptic coupling parameters were scaled by 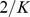.

To make explicit the role of the balanced state in generating selectivity, we investigated a network, which is obtained by scaling all synaptic weights of the network by 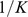 rather than 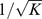. Thus, in this network, both the net excitatory and inhibitory potentials are of the order of the spike threshold, hence there is no pressure for balancing excitation and inhibition. Contrary to the balanced state, the spiking in this network are regular, as quantified by low CV values of ISI distributions (Fig. 8A). For the example parameters, excitatory neurons fire with a mean rate 9.6 Hz and mean C.V 0.36. Inhibitory neurons fire at 9.1 Hz with a mean CV 0.49. Both selectivity and sparseness fall as 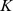 increases (Fig. 8B). Due to the weak scaling of synaptic weights, now the variation in input current is 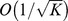 relative to the mean current, which vanishes in the infinite 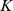 limit. Selectivity and sparseness fall as stimulus intensity increases (Fig. 8C), as expected from the ‘iceberg effect’ caused by spike threshold nonlinearity. This is in contrast to the balanced network, where we showed that as the stimulus intensity increases, selectivity and sparseness increase, except at very low rates. Finally, in our networks with weak synapses, for the set of parameters we investigated in this paper, the paradoxical behavior was not observed. For example, elimination of all external input to inhibitory neurons caused a large increase in excitatory population rate from 12 Hz to 35 Hz but decreased the inhibitory population activity from 14 Hz to 11 Hz.

## Discussion

We showed that randomly connected networks can generate and maintain stimulus selectivity and population sparseness robustly in the balanced regime. In randomly connected networks, stimulus-to-stimulus and neuron-to-neuron variations in the synaptic input are small compared to the untuned component of the synaptic input. Balanced networks solve this problem by using strong synapses to amplify the variation in synaptic input and using recurrent inhibition to cancel the untuned mean. Moreover, selectivity and sparseness are robust to changes in the connection density. An unbalanced network, on the other hand, can show sharp selectivity at some low connection densities due to thresholding, but selectivity rapidly decreases with increasing connection density due to decrease in stimulus-to-stimulus variation in the synaptic input. Sparseness in the unbalanced network follows the same trend.

A well-known hypothesis regarding cortical connectivity (‘fire together wire together’) is that excitatory neurons with similar response properties are more likely to be connected (or their connections are stronger) than pairs with dissimilar response properties. In this paper, we studied networks with random connectivity, where the term ‘random connectivity’ is used to characterize the generative statistical rule used to build the network, namely connections between neuronal pairs are drawn from a statistical distribution that is independent of any stimulus related feature. Naively, a randomly connected network should not exhibit preferential connectivity between neurons with similar response properties. However, in our network we observed a correlation between connection probability and response similarity. Excitatory neurons with similar preferred stimuli connect with a higher probability to each other. Note that this connection probability is conditioned on response similarity, and is a different quantity than the connection probability that was used to generate the network. Hence, functional specificity emerged even if the network was randomly connected and it is the heterogeneity in connectivity caused by the random generative statistical rule that lead to this result. Then, observation of functional specificity is not sufficient to claim non-random connectivity. It is the magnitude of this specificity that distinguishes randomly connected networks. We quantified this magnitude by the CMI and showed that CMI varies significantly with the mean number of synapses a neuron receives, 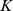.

Fig. 2D shows that at low 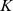 the CMI of the feedforward excitatory connections (i.e., 0->E) is large in our random network. This is understandable, given that information about the stimulus in the output layer comes from the stimulus selectivity of the input layer. The CMI of the recurrent connections within the output layer are much weaker but can nevertheless be significant in low 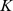. Since the recurrent connections are drawn independently from the input connections, the residual CMI of the recurrent connections reflect the fact that the stimulus preference of the output of each neuron is the combined effect of the stimulus modulation in the three synaptic inputs, the 0->E, the E->E and the I->E. Thus, although the stimulus preference at the output neurons is largely determined by the input from the input layer, they are biased also by the selectivity of the recurrent sources. The weak modulation of the output preference by the recurrent excitatory inputs implies that the functional role of the recurrent excitation is mostly to increase the drive of the neurons by providing essentially untuned excitation. Furthermore, the magnitude of the modulation of connectivity in all three pathways falls off strongly with increasing the mean number of connections, despite the fact that the firing rate selectivity is insensitive to increased 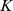. This dissociation between the strength of the synaptic tuning and the sharpness of the spiking output is the hallmark of the balanced network.

The existence of orientation selectivity in visual cortices with “salt-and-pepper” architecture [4], [48], [49], and the observation of [50] that layer 2/3 neurons in mice visual cortices receive input from a set of neurons with a wide range of preferred orientations motivated a randomly connected balanced network model, similar to the present one [33], [37]. However, the lack of feature maps in visual cortex does not necessarily imply that there is no functionally specific connectivity or that the network is randomly connected. It was observed in recent experiments in adult mice [45], [51] (but not in young; [30]) that pyramidal neurons with similar preferred orientations are twice more likely to connect to each other than pyramidal neurons with orthogonal preferred orientations. Our analysis indicates that such a strong modulation of recurrent excitatory connections is inconsistent with the weak modulation expected in randomly connected networks even in the balanced state. Thus, these experiments suggest that despite the lack of columnar organization for orientation, the recurrent connectivity is tuned by orientation similarity by a non-random mechanism, which could be Hebbian in origin.

In our networks, the stimulus selectivity of the output neurons is biased away from the stimulus preference of the inhibitory inputs. Thus, neurons in the balanced network are likely to receive peak excitation at their preferred stimulus, while peak inhibition is more likely to be received at the least preferred stimulus (Fig. 2C). This is in contrast to balanced network models in which recurrent connections are constructed as stimulus specific [13], [14]. In these networks both excitatory input and inhibitory input would show peaks at or near the postsynaptic cell’s preferred stimulus. The reason for this is that the strong tuning of the recurrent excitatory input must be balanced (i.e. cancelled away) by similarly tuned inhibition. This is not the case in our model where the tuning of the excitatory input is relatively weak.

Random connectivity leads to Gaussianity of the distribution of synaptic inputs to neurons. This in turn means that only first two moments of the input layer activation statistics are important for the balanced network’s operation. We varied these statistics systematically. We showed that as the stimulus intensity (mean firing rate of input layer neurons) increases, selectivity and sparseness increase, except at very low rates. In the unbalanced network, both selectivity and sparseness decrease sharply with increasing stimulus intensity due to the “iceberg” effect. Varying the second order statistic led us to conclude that selectivity and sparseness increase with sparseness of the input. An interesting observation was that when the input population was uniformly responding to all stimuli (its SPI and SLI were zero), the balanced network continued to produce strong sparseness while the selectivity was zero (Fig.s 4C and D). Selectivity generated in the random connected network is ultimately due to selectivity in the input. Sparseness, on the other hand, is generated largely by the heterogeneity among neurons in the number of synapses they receive. We showed that if one were to set up the random balanced network constraining the number of synapses that a neuron receives to be strictly 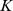, as in [52], sparseness would too vanish for uniform input.

Network selectivity is modestly affected by changes in synaptic weights. We observed that inhibitory activity is the main factor that affects network selectivity when excitatory activity was kept fixed (Fig.s 6A and B). Overall scaling of synaptic weights did lead to a monotonic increase in selectivity while the changes in network activity were small.

An interesting finding was that decreasing membrane time constants led to a monotonic decrease of selectivity. This trend is worth exploring further, since in the more realistic conductance-based neuron models effective membrane time constants are known to be small. It will be interesting to see if similar levels of selectivity and sparseness can be achieved in balanced networks with conductance-based neurons [13], [53], [54].

In the olfactory cortex odor mixtures cause suppression, that is a neuron responsive to one but not to another odor will show a decreased response when presented with both odors [23]. Cross-orientation suppression seen in visual cortices is a similar phenomenon: responses of V1 neurons to preferred orientations are suppressed by superimposed gratings of orthogonal orientations [55], [56]. The balanced network shows a suppression effect, purely due to recurrent interactions in the network, as the input layer response in our model is additive.

Recent studies in mouse visual cortex [57]–[59] found that optogenetic silencing or activation of inhibitory neurons caused an increase or decrease respectively in the excitatory network activity rate. In balanced networks, whether connectivity is tuned or not, a change in excitatory input to inhibitory neurons would change the excitatory and inhibitory population firing rates in the same direction. In particular, as in other inhibition-stabilized networks [46], [47], [60], a decrease in excitatory drive to inhibition caused increased network firing rate for both populations (an effect termed “paradoxical” [46]). The unbalanced network however, does behave as observed in experiments. Furthermore, [57] found that when interneurons are suppressed, which lead to increased pyramidal cell population firing rate, visually evoked inhibitory postsynaptic conductance is reduced in pyramidal cells while visually evoked excitatory conductance did not change. In contrast, balanced networks would require both to track each other.

In the random networks we considered, inhibitory neurons are almost as selective as excitatory ones. Experiments report both selective and unselective interneurons in the sensory cortex. Olfactory cortex neurons are broadly tuned and provide global inhibition [7], [44]. In mouse visual cortex, somatostatin-expressing interneurons are as selective as pyramidal cells [43], but parvalbumin-expressing (PV) interneurons have low selectivity [43], [45]. Potential explanation for the latter behavior comes from the finding that local connectivity from pyramidal cells to PV interneurons is dense, that is the probability of a pyramidal cell making a synapse to a nearby PV interneuron is almost 1 [5]. Combined with the salt-and-pepper architecture of mouse visual cortex, this means that within layer excitatory synaptic input to PV interneurons is almost untuned. In our model, by making both within layer and feedforward excitatory synapses to inhibitory neurons dense, we could achieve a balanced state and get low inhibitory neuron selectivity (Fig. 5C). It remains to be seen if other types of input to PV interneurons are also untuned.

In the models we presented, selective and sparse response results from the heterogeneity in the number of synapses a neuron receives. Real neural networks exhibit many other sources of heterogeneity. For example, synapses of a given type do not have the same strength but are lognormal distributed [61] and the single-cells of a population differ in their physiological properties. Our preliminary observations (data not shown) suggest that both types of inhomogeneity (the latter being modeled as variability in neuron thresholds) increase selectivity and sparseness in the network. It would be interesting to study more conclusively the effect of such heterogeneities.

In conclusion, the ‘salt and pepper’ architecture of several cortical networks, particularly in rodents, suggest that stimulus selectivity can be sustained even when local cortical connectivity is poorly tuned to stimulus selectivity, as is the case in the random balanced networks. Alternatively, activity dependent plasticity may induce strong stimulus selectivity in the recurrent connections without any topographic order. At present the experimental support for this paradigm is ambivalent. In mouse visual cortex, strong orientation tuning of layer 2/3 local recurrent excitatory connections exists in the adult animal but not at eye opening despite the early presence of sharp tuning. On the other hand, at eye opening, there seems to be a weak modulation on the recurrent connections as a function of signal correlations when the animal is presented with natural movies [30]. This might be a sign of structure in connectivity that is independent of orientation tuning. Networks with such structure are beyond the scope of this paper. In addition, currently, the degree of selectivity in the feedforward input (from LGN or layer 4) to layer 2/3 neurons in mouse visual cortex is unknown. In olfaction, recent experiments indicate that the projections from the olfactory bulb to the piriform cortex in mouse are random [24]–[29]. It would be interesting to know whether the recurrent connections in piriform cortex are also poorly tuned.
